# A case of secondary syphilis manifesting as a pulmonary pseudo-tumour with nephrotic syndrome

**DOI:** 10.7196/AJTCCM.2021.v27i2.065

**Published:** 2021-06-23

**Authors:** C B I Coccia, E Makambwa, C N Jackson, D R Chetty, Q Said-Hartley, G Symons

**Affiliations:** 1 Departments of Medicine, Faculty of Health Sciences, University of Cape Town and Groote Schuur Hospital, Cape Town, South Africa; 2 Department of Pathology, Division of Anatomical Pathology, Faculty of Health Sciences, University of Cape Town and Groote Schuur Hospital, Cape Town, South Africa; 3 Department of Radiology, Faculty of Health Sciences, University of Cape Town and Groote Schuur Hospital, Cape Town, South Africa

**Keywords:** syphilis, pulmonary syphilis, nephrotic syndrome

## Abstract

The global incidence of primary and secondary syphilis is increasing in high-risk groups. However, pulmonary syphilis remains exceedingly rare with less than 30 cases recorded since 1967. Of these cases, none have recorded the presence of both pulmonary and renal involvement with nephrotic syndrome. Diagnosis of pulmonary syphilis remains a challenge, and there is no consensus on treatment. We report a case of a 46-year-old male with secondary pulmonary syphilis and concomitant nephrotic syndrome.

## Background

Syphilis, a sexually transmitted disease caused by *Treponema pallidum*, is most prevalent in Africa.^[1]^ However, its incidence has been decreasing. In contrast, the incidence has been rising in high-risk groups outside Africa.^[2]^

Despite the increase in incidence globally, pulmonary syphilis remains exceedingly rare. Historically, pulmonary syphilis was seen in congenital and tertiary syphilis (as gummas) in the pre-antibiotic era but close to 30 cases have since been described since 1967.

We present a case of secondary syphilis with both pulmonary involvement and nephrotic syndrome that was initially thought to be metastatic lung cancer.

## Case

A 46-year-old male presented to the emergency unit with a 3-month history of progressive lower limb swelling, a persistent non-productive cough and non-specific abdominal discomfort that had worsened over the preceding week. Other than a previous traumatic lower limb amputation, he reported no medical comorbidities but had a significant smoking history. On initial assessment the patient was afebrile, had significant pedal oedema and sub-centimeter inguinal lymph nodes with no visible or reported skin rashes. Cardiovascular, respiratory and neurological examinations were normal. Minimal abdominal tenderness was elicited, and he was noted to have extensive penile ulceration.

Initial urine dipsticks revealed significant proteinuria therefore a cause for nephrotic syndrome was investigated. Notable preliminary results included a urine protein creatinine ratio (U-PCR) of 0.317 g/mmol, normal renal function (creatinine of 58 µmol/L), a raised calcium (3.12 mmol/L) and a cholestatic liver profile (alanine transaminase of 33 U/L, gamma-glutamyl transferase of 349 U/L, and alkaline phosphatase of 461 U/L). His serology was negative for HIV and hepatitis B, but positive for *T. pallidum* antibody (TPAb). The TPAb antibody assay is an initial screening in our laboratory and if positive, a rapid plasmin reagin (RPR) gets performed. The RPR results became available 72 hours after admission, by that time the patient had already undergone chest radiography and computed tomography (CT).

**Fig. 1:**
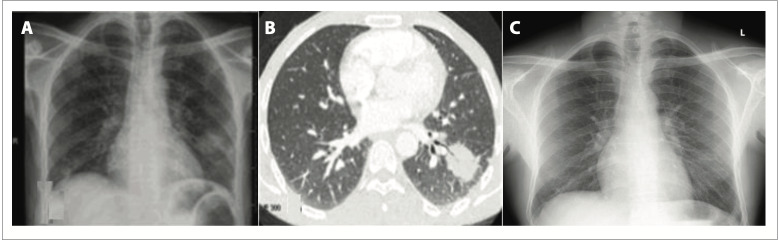
Initial chest X-ray imaging showing left lower lobe mass with smaller pulmonary nodules (A); contrasted chest computed tomography showing irregular left lower lobe mass measuring 37 × 38 × 26 mm (B); and chest X-ray 8 weeks post-treatment (C).

Admission chest radiography revealed a suspicious left lower lobe mass with multiple smaller pulmonary nodules in the lower zones (Fig. 1A). Contrasted chest CT was performed the following day, which showed an irregular left lower lobe mass (37 × 28 × 26 mm) with multiple posterior lower lobe subpleural nodules, sub-centimetre mediastinal and bilateral hilar nodes, no pleural effusions and background paraseptal emphysematous changes in the upper lobes (Fig. 1B).

In view of the patient history, blood results (raised calcium and liver enzymes), U-PCR and CT findings, the provisional diagnosis was that of metastatic lung cancer complicated by nephrotic syndrome. The patient subsequently underwent CT-guided biopsy of the lung lesion. In the interim, the result of RPR came back positive with a titre of 1:256 – this was thought to be an independent finding and unrelated to the current clinical presentation.

Provisional histology of the lung mass reported no evidence of atypia or malignancy. There was a marked plasma infiltrate with multiple granulomas, and some showed early central necrosis. Furthermore, scattered anthracotic pigment deposition was noted within the stroma. There were no acid-fast bacilli identified after Ziehl-Neelsen and modified Ziehl- Neelsen staining or fungi after Periodic Acid-Schiff and Grocott staining. Gram stain showed no organisms and subsequent bacterial culture was negative. In view of these findings and positive RPR, further immunohistochemistry was requested. AE1/3 highlighted entrapped reactive alveolar structures and *T. pallidum* antibody was positive. The granulomatous inflammation and positive TPAb supported a final diagnosis of pulmonary syphilis with early necrotic granulomas being precursors of gummas.

The patient went on to have a renal biopsy, which showed mild mesangial proliferative glomerulonephritis, keeping with syphilitic nephropathy. Renal ultrasound showed an oedematous right kidney with moderate loss of cortico-medullary differentiation in both kidneys. Although no formal imaging or tissue sampling of the liver was done, the raised canalicular enzymes suggested liver involvement.

The patient was treated with two doses of benzyl-penicillin (2.4 million U) intramuscularly 2 weeks apart and received supportive therapy in the form of an angiotensin-converting enzyme inhibitor, a loop diuretic and paracetamol for analgesia. He responded well with no signs of a Jarish-Herxeimer reaction. His symptoms resolved within a few days of commencing therapy and ten days later, his U-PCR improved from 0.317 g/mmol to 0.049 g/mmol. Chest radiography revealed that the left lung mass had resolved at 8 weeks of follow-up (Fig. 1C), the RPR titre decreased to 1:64, and normalisation of serum calcium and liver function.

## Discussion

Syphilis, also known as ‘The Great Imitator’, was first described in 1530 by Hieronymus Fracastorius. The association with *T. pallidum* was later made in 1905 by Schaudinn and Hoffman^[3]^ when they identified the bacteria in syphilitic lesions.

Secondary syphilis typically occurs 6 - 8 weeks after spontaneous resolution of the primary chancre. It may however occur several months later, or not at all. Common systemic manifestations of secondary syphilis may include malaise, fever, anorexia and weight loss. Mucocutaneous involvement occurs in 80% of patients. The mucous membrane lesions are highly infectious and may recur without treatment. Less frequently, neurological, gastrointestinal, renal or pulmonary complications may arise.

Neurological sequelae include acute meningitis, sensorineural hearing loss, Bell’s palsy, uveitis and iritis. Gastrointestinal involvement includes syphilitic gastropathy, proctitis and hepatitis with hepatomegaly. Although severe hepatitis with right upper quadrant pain may occur, it is usually mild with no jaundice, a slight increase in bilirubin and gamma-glutamyl transferase, and a disproportionate increase in alkaline phosphatase.^[4]^ Renal manifestations include mild albuminaemia, membranous glomerulonephritits, mesangial and epithelial cell proliferative glomerulonephritis, rapidly progressive cresenteric glomerulonephritits, and minimal change nephrotic syndrome with acute renal failure.^[5]^ Pulmonary manifestations may be asymptomatic, or may present as pleurisy or bronchitis. Chest X-ray typically shows multiple pulmonary nodules at the lung bases; however, may show larger masses or a pleural effusion.

For the diagnosis of pulmonary syphilis, the following criteria should be fulfilled: (i) history and clinical findings typical of secondary syphilis; (ii) serological testing positive for secondary syphilis; (iii) pulmonary abnormalities seen on radiographs, with or without associated symptoms; (iv) exclusion of other pulmonary disease by serological tests, sputum smear, cultures and sputum cytology; and (v) therapeutic response of the radiological findings to anti-syphilitic therapy.^[6]^

Pulmonary syphilis is exceedingly rare. In the 1910s and 1920s, multiple autopsies were conducted in numerous institutes supporting this statement, with overall pulmonary involvement in that era only being evident in 1 - 12% of autopsies in patients known to have syphilis.^[6]^ Since 1967, there have been close to 30 reported cases of secondary pulmonary syphilis. Of these cases, only one reported renal involvement in the form of sub-nephrotic range proteinuria. Furthermore, renal syphilis with concomitant hepatic involvement in itself is also exceptionally rare with only seven cases described in the literature.^[3]^ The case we report not only fulfills the five criteria for diagnosis of pulmonary syphilis, but to our knowledge, illustrates the first case of pulmonary syphilis with nephrotic syndrome. Additionally, the raised liver enzymes suggest concomitant liver involvement – adding to the uniqueness of this case.

Although syphilis is a seemingly historical disease, this case report reiterates the importance of recognising its various clinical presentations and maintaining it as a differential diagnosis to achieve timeous treatment and prevent long-term complications.
